# Impedimetric Aptasensor for Ochratoxin A Determination Based on Au Nanoparticles Stabilized with Hyper-Branched Polymer

**DOI:** 10.3390/s131216129

**Published:** 2013-11-26

**Authors:** Gennady Evtugyn, Anna Porfireva, Veronika Stepanova, Marianna Kutyreva, Alfiya Gataulina, Nikolay Ulakhovich, Vladimir Evtugyn, Tibor Hianik

**Affiliations:** 1 Analytical Chemistry Department, Kazan Federal University, 18 Kremlevskaya Street, Kazan 420008, Russian Federation; E-Mails: Gennady.Evtugyn@kpfu.ru (G.E.); porfireva-a@inbox.ru (A.P.); caosao@mail.ru (V.S.); 2 Inorganic Chemistry Department, Kazan Federal University, 18 Kremlevskaya Street, Kazan 420008, Russian Federation; E-Mails: mkutyreva@mail.ru (M.K.); alphiag@mail.ru (A.G.); Nikolay.Ulakhovich@kpfu.ru (N.U.); 3 Electron Microscopy Laboratory of the Faculty of Biology, Kazan Federal University, 18 Kremlevskaya Street, Kazan 420008, Russian Federation; E-Mail: vevtugyn@gmail.com; 4 Department of Nuclear Physics and Biophysics, Comenius University, Mlynska dolina F1, Bratislava 842 48, Slovakia

**Keywords:** aptasensor, ochratoxin A, DNA aptamers, Au nanoparticles, hyper-branched polymer

## Abstract

An impedimetric aptasensor for ochratoxin A (OTA) detection has been developed on the base of a gold electrode covered with a new modifier consisting of electropolymerized Neutral Red and a mixture of Au nanoparticles suspended in the dendrimeric polymer Botlorn H30^®^. Thiolated aptamer specific to OTA was covalently attached to Au nanoparticles via Au-S bonding. The interaction of the aptamer with OTA induced the conformational switch of the aptamer from linear to guanine quadruplex form followed by consolidation of the surface layer and an increase of the charge transfer resistance. The aptasensor makes it possible to detect from 0.1 to 100 nM of OTA (limit of detection: 0.02 nM) in the presence of at least 50 fold excess of ochratoxin B. The applicability of the aptasensor for real sample assay was confirmed by testing spiked beer samples. The recovery of 2 nM OTA was found to be 70% for light beer and 78% for dark beer.

## 1. Introduction

**Figure 1.**
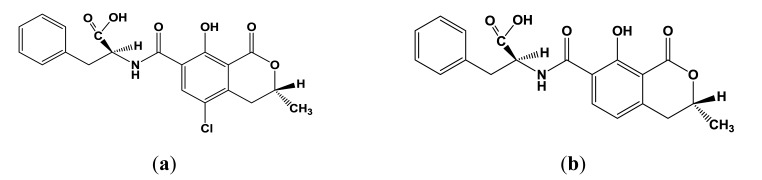
Structures of ochratoxin A (**a**) and ochratoxin B (**b**).

Ochratoxin A (OTA, Figure 1a) is a secondary metabolite produced by filamentous fungi of the genera *Aspergillus* and *Penicillium* present in a many foodstuffs, e.g., cereals, beans, coffee, cocoa, grapes and spices [1,2]. The high stability of the OTA, especially toward high temperatures, results in contamination of processed foods, e.g., cereal products, instant coffee, dried fruits, bread, beer and wine [3–7]. OTA exerts nephrotoxic, carcinogenic, teratogenic, immunotoxic and hepatotoxic effects and can probably cause nephropathies and urothelial tumours in humans [8–11]. Ochratoxin A is the most prevalent and relevant fungal toxin of this group, while ochratoxin B (Figure 1b) and C (ethyl ester of OTA) are of lesser importance.

The Joint FAO/WHO Expert Committee on Food Additives (JECFA) evaluated a provisional tolerable weekly intake of OTA equal to 112 ng/kg of body weight per week (1991) [12]. More recently, the following maximal admissible levels have been established for OTA by European Commission: 3 μg/kg (7.4 nM) for cereal products, 5 μg/kg (12.4 nM) for roasted coffee, 10 μg/kg (25 nM) for dry grapes [13,14], and 0.5 μg/kg (1.2 nM) for all baby food [13].

OTA contamination is commonly detected by HPLC with fluorescent [15,16] or mass spectroscopy [17,18] detection coupled with alkaline or solid-phase extraction [19]. From other methods, immunochemical detection with SPR [20,21], electrochemical [21–25] and fluorescence [26–28] sensing can be mentioned (see also review [29] on immunochemical OTA detection and references cited). Direct oxidation of OTA from alkaline solution on glassy carbon electrode was evaluated by square-wave voltammetry [30].

Being very sensitive and reliable, immunoassay techniques require time- and labor-consuming stages of antibody isolation, their purification and modification. Measurement commonly involves a number of incubation/washing stages which increase the duration of the assay and accumulate errors related to insufficient stability of components, their losses and degradation.

DNA/RNA aptamers are synthetic oligonucleotides able to bind target analytes with high specificity and efficiency [31]. The interest toward aptamers as biorecognition elements is related to their rather simple synthesis strategy (SELEX technology [32,33]) and promising opportunities of their application in biosensors, electrophoresis and affine chromatography [34]. In comparison with antibodies, aptamers show higher stability, easier operation mode and better reproducibility of binding properties in production and storage period. The modification of the aptamers with optical or redox labels as well as introduction of terminal functional groups required for immobilization are easier than similar modification of antibodies [35].

Au nanoparticles have been attracting increasing attention in the biosensor development field during the past decades [36,37]. This is mainly related to the ability of gold nanoparticles to provide a stable immobilization of biomolecules via their attachment to the metal surface by thiol groups. Furthermore, Au nanoparticles permit direct electron transfer between redox proteins and electrode, allowing electrochemical sensing with no need of mediators. A number of electrochemical aptasensors with implementation of Au nanoparticles have been described for OTA detection. Thus, dual labeling of aptamers with Au nanoparticles and methylene blue was employed for signal amplification. The formation of the aptamer-OTA complex switched the conformation of an aptamer so that methylene blue was approached to the electrode surface and involved in the electron exchange yielding the voltammetric response [38]. Salt-induced aggregation of Au nanoparticles was applied for colorimetric detection of OTA-aptamer interactions [39]. Au nanoparticles were modified with *N*-(aminobutyl)-*N*-ethylisoluminol as luminescent label for electrochemiluminescent OTA detection based on the displacement protocol [40]. Some other examples of the use of aptamers in the assembly of electrochemical and electrochemiluminescent aptasensors for the detection of various analytes are considered in [41] with particular emphasis to the surface functionalization and measurement protocol applied.

The performance of such aptasensors as well as other bioanalytical applications of Au nanoparticles depend on the efficiency of stabilization of nanoparticles and their entrapment in the surface layer allowing access of biorecognition elements and analyte molecules. Hyper-branched polymers have found increasing application as nanoreactors and stabilizers of metal dispersions due to variety of functional groups implemented in terminal substituents and possibility of direct control of hydrophobic/hydrophilic balance of the aggregates produced. Thus, Ag, Au, Pt, Pd and Cu nanoparticles have been obtained in the presence of polyethylene imines [42,43], polyamidoamines [44,45], polyaminoesters [46] and aromatic polyamides [47,48]. Hyper-branched polymers bearing chelating dithiocarbamates were successfully applied for the synthesis of the Au nanoparticles [49].

The hyper-branched polyesterpolyols Boltorn H^®^ and their derivatives are used as homogeneous nanoreactors for the synthesis of metal nanoparticles, Thus, the nanocrystals of PbS were obtained in the Boltorn H^®^ surrounding and characterized with IR spectroscopy, X-ray diffraction, photo-luminescence and transmission electron microscopy (TEM) [50]. Ag nanoparticles were also obtained in similar experimental conditions [51]. As was mentioned, the use of Boltorn H^®^ provided the formation of well-shaped particles with uniform size and spatial distribution in the matrix. What is important for biosensor development, amphiphilic Boltorn molecules are biocompatible and were used as drug carriers for the delivery of 5-fluorouracil and doxorubicin [52,53]. In this work, Boltorn H^®^ stabilized Au nanoparticles were applied for the development of an OTA aptasensor.

## 2. Experimental Section

### 2.1. Reagents

**Figure 2.**
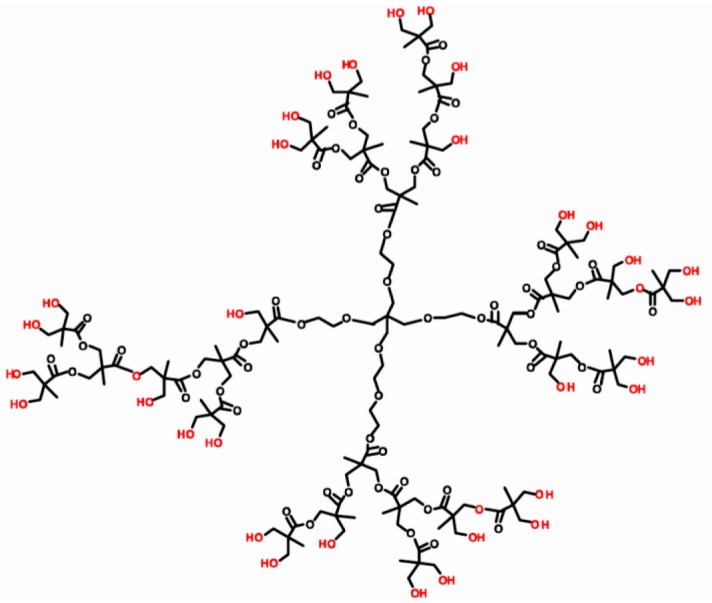
The structure of Boltorn H30^®^ applied for the stabilization of Au nanoaprticles and aptasensor development.

The DNA aptamer 5′-SH-GAT CGG GTG TGG GTG GCG TAA AGG GAG CAT CGG ACA-3′ specific to OTA was designed according to [54] and purchased from Thermo Fisher Scientific GmbH (Ulm, Germany). Gold (III) chloride hydrate (HAuCl_4_·H_2_O), OTA (M.w. 403.8 Da), ochratoxin B, neutral red (NR), HEPES ((4-(2-hydroxyethyl)-1-piperazineethanesulfonic acid) were purchased from Sigma-Aldrich (St.Louis, MO, USA), ethanol (99%) and hydrazine hydrate (99%) from Acros Organics (Geel, Belgium). Hyper-branched polyesterpolyol based on 2,2-dihydroxypropane acid Boltorn H30 (32 hydroxy groups, M.w. 3,560, hydroxyl number 480–520 mg/g KOH) was purchased from Perstorp Speciality Chemicals AB (Perstorp, Sweden). The structure of Boltorn H30^®^ is presented in Figure 2.

All the reagents were of analytical grade and used without additional purification. Millipore Q^®^ water was used for preparation of working solutions.

### 2.2. Apparatus

Electrochemical measurements were performed with the AUTOLAB PGSTAT 302N potentiostat with FRA module for electrochemical impedance (EIS) measurements (Metrohm Autolab b.v., Utrecht, The Netherlands). Three-electrode cell with Au working electrode (Φ = 2 mm), Pt auxiliary electrode and double junction Ag/AgCl reference electrode (Autolab) were used for all the electrochemical measurements performed in DC mode.

**Figure 3.**
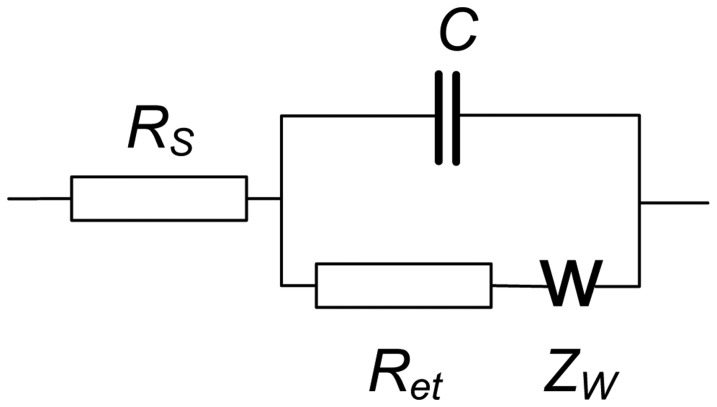
Randles' equivalent circuit applied in EIS measurements.

EIS spectra were recorded using the NOVA software of the PGSTAT 302N in the presence of 0.05 M K_3_ [Fe(CN)_6_] and 0.05 M K_4_ [Fe(CN)_6_]. The amplitude of the applied sine potential was 5 mV. The direct current potential was calculated as half-sum of the peak potentials of the redox couple [Fe(CN)_6_] ^3−/4−^. The EIS spectra were recorded within the frequency from 100 kHz to 0.04 Hz with a sampling rate of 12 points per decade. The calculations of capacitance and resistance from EIS spectra were made using fitting procedure corresponding to the Randles' equivalent circuit shown in Figure 3.

Here *R_S_* and *R_et_* are the electrolyte and electron transfer resistances, *Z_W_* is the Warburg impedance and *C* the capacitance of the electrode surface/solution interface. The dimensionless index *n* (roughness factor) was higher than 0.85 in all the experiments, so that pure capacitance *C* can be used instead of the constant phase angle element expressing non-ideal capacitive response of the interface. The *R_et_* and *C* changes reflect the interaction of aptamer with OTA at the electrode surface whereas the *R_S_* was quite constant.

TEM images were recorded with Jeol JEM 100 CX II transmission electron microscope (Tokyo, Japan) operating at 80 kV. The samples were mounted on formvar-coated 150-mesh nickel grid.

FTIR spectra were recorded on Spectrum 400 (Perkin Elmer, Waltham, MA, USA) in the range from 4,000 to 400 cm^−1^. Electron absorbance spectra were obtained with Lambda 750 spectrophotometer (Perkin Elmer) at 190–900 nm in thermostated (25 ± 0.01 °C) 1 cm quartz cuvette equipped with heating circulator with bath Julabo MB-5A (Julabo, Seelbach, Germany) thermostat and Peltier thermostat PTP-1 (Perkin Elmer).

### 2.3. Synthesis of Au Nanoparticles

Boltorn H30^®^ (0.71 g) were dissolved under heating to 60 °C in aqueous ethanol (1:1, 3 mL), then the solution was cooled to ambient temperature. After that, AuCl_3_ (9 mg) were added and the resulting solution mechanically stirred for 10 min. Hydrazine hydrate (N_2_H_4_·H_2_O, 30 mg) was added dropwise to form a deep blue color. The resulting solution was stored at 4 °C for not more than one week and diluted in the 1:5 ratio with 0.05 M HEPEs buffer solution containing 0.1 M KCl prior to deposition on the electrode surface.

### 2.4. Preparation of Aptasensor and OTA Determination

The Au electrode was first mechanically polished by alumina and then ultrasonicated in 0.1 M sulfuric acid and deionized water. After that, it was electrochemically cleaned by repeated cycling of the potential in 0.2 M H_2_SO_4_ solution in the range from −0.5 to 0.8 V until the background current stabilized. After that the electrode was transferred in the 0.025 M phosphate buffer solution containing 0.1 M KNO_3_ and 3.46 mM NR, pH = 6.0. The electropolymerization was performed by multiple cycling the potential from −0.8 to 0.8 V with the scan rate of 50 mV/s followed by electrostatic polarization at 0.8 V for 5 min. After that, the electrode was rinsed with Millipore water and dried at ambient temperature. The deposition of Au nanoparticles was performed by casting 1 μL of the mixture obtained by chemical reduction of Au in the presence of Boltorn H30^®^. Then the electrode was fixed upside down and 5 μL of 1.25 μM thiolated aptamer solution were placed onto the working surface. The electrode was capped with plastic tube to prevent its drying. After 15 min incubation, the electrode was washed with distilled water and placed in electrochemical cell containing 4.5 mL of 0.05 M HEPES buffer solution containing 0.1 M KCl, pH = 7.0. The EIS measurements were performed in the presence of 0.05 M K_3_ [Fe(CN)_6_] and 0.05 M K_4_ [Fe(CN)_6_] three times with intermediate magnetic stirring of the solution.

For OTA measurement, the aptasensor was fixed upside down and 10 μL of OTA were placed on its surface. For the incubation period (30 min) the aptasensor was capped with plastic tube, then washed with Millipore water and twice with HEPES buffer solution and the EIS measurements was performed as described above.

## 3. Results and Discussion

### 3.1. Modification of the Electrode with Au/Boltorn H30^®^ Suspension

**Figure 4.**
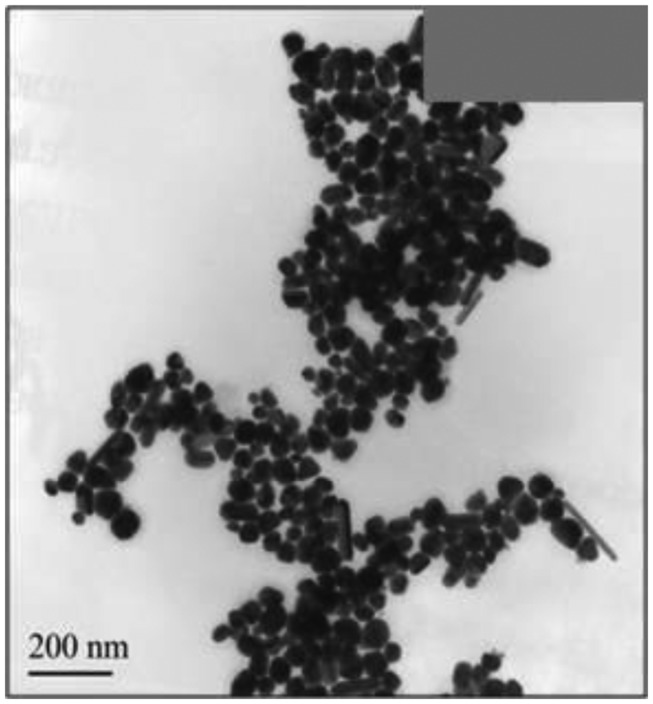
TEM images of Au/Boltorn H30^®^ suspension obtained on Ni grid (150 mesh).

Chemical reduction of AuCl_4_^−^ has been performed in the presence of Boltorn H30^®^ which stabilized the suspension and limited the growth of the Au nanoparticles. The use of hydrazine as reducer made it possible to avoid possible contamination of the suspension with the products of the reaction. The formation of Au nanoparticles as well as working conditions of the reduction were investigated using TEM, FTIR and IR spectroscopy. The UV-vis spectra of the suspension obtained in the above mentioned conditions (see Section 2.3) contained a broad absorption peak with maximum at 530 nm corresponded to the average diameter of the Au nanoparticles of 40–80 nm [55]. The size distribution was confirmed by TEM indicating the formation of well-defined granulated rounded particles amalgamated in elongated filamentous aggregates (Figure 4)

The participation of polymer molecules in the stabilization of Au nanoparticles was confirmed by FTIR spectroscopy. The formation of Au nanoparticles was followed by an additional band at 1,648 cm^−1^ corresponding to valence vibrations of >C=O groups of esteric fragments of the polymer. This indicates localization of the Au nanoparticles near esteric groups of the dendrimer.

The stability and size distribution of the Au nanoparticles did not change dramatically with the concentration of hydrazine added in a high molar excess and after a long reaction time. The reaction was monitored by a color changes and stopped after reaching a deep blue color. The decrease of the concentration of stabilizer below a w/w ratio of Boltorn H30^®^:AuCl_3_ = 100:1 resulted in the amalgamation of the metal particles and precipitation of a solid sediment within a several hours after the suspension preparation. In optimal conditions, the Au/Boltorn H30^®^ suspension retained the size distribution of the particles and sedimentation stability for more than two weeks when stored at 4 °C. Meanwhile, from the considerations of the reproducibility and stability of the modifier layers onto the electrode, the storage period of Au/Boltorn H30^®^ suspensions was limited with one week for the aptasensor preparation.

The modification of the Au electrode with Au/Boltorn H30^®^ suspension was performed by drop casting of the aliquot followed by drying at ambient temperature. It was shown that the electrode modified with Au nanoparticles did not exert any redox activity within the potential window from −0.8 to 0.8 V. In blank experiments, an equivalent quantity of Boltorn H30^®^ was added. The presence of Au nanoparticles slightly suppressed the background currents and shifted the discharge of the electrolyte to higher anodic potentials by 200–250 mV. The periodic recording of the cyclic voltammograms did not show any changes in the morphology of the voltammograms within 10 h of continuous operation. For this period of time, no changes in the color and uniformity of the modifying film were found.

### 3.2. Electrochemical Characterization of the Au/Boltorn H30^®^ Suspension on the Electrode Surface

**Figure 5.**
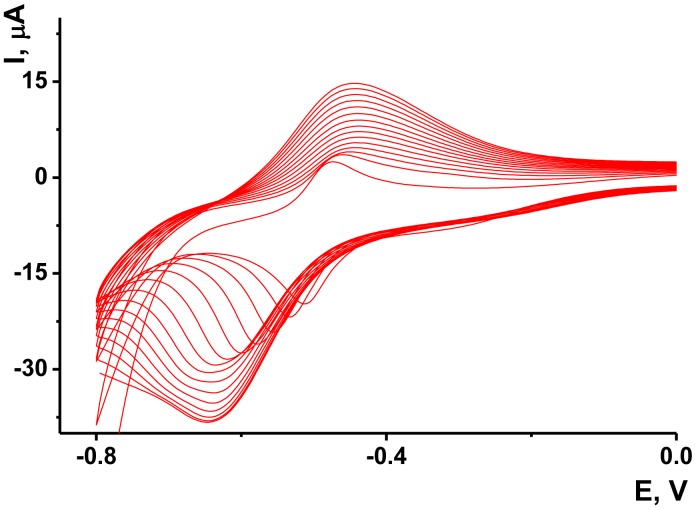
Cyclic voltammograms recorded at the scan rate of 50 mV/s on the Au electrode in 3.46 mM NR solution in 0.025 M phosphate buffer, pH = 6.0.

As no redox activity was observed for the Au/Boltorn H30^®^ suspension, the conditions for the electron transfer were studied using ferricyanide ion as redox indicator. Preliminary experiments performed with freshly deposited Au/Boltorn H30^®^ suspension showed instability of the response toward 1.0 mM [Fe(CN)_6_] ^3−^, caused probably by irreversible changes of the film coating. To stabilize the signal, Au electrode was polarized at 800 mV. Nevertheless, the redox peaks of ferricyanide ions decayed in multiple cycling of the potential while the difference of the peak potential increased to about 200 mV against 120 mV for the first cycle. This could be because of the shielding influence of stabilizer, which prevents direct contact of Au nanoparticles with the transducer surface. Previously it was shown for similar modifier with sterically hindered redox centers that the conditions of electron transduction can be significantly improved by underlying the primary layer with a thin film of electropolymerized material [56]. NR, a phenazine dye, showed excellent transduction properties being polymerized onto glassy carbon by multiple potential cycling within −800 and 1,100 mV. Following the conditions determined for this sensor, we deposited polymeric NR form on the Au electrode prior to Au/Boltorn H30^®^ casting. The voltammograms obtained in NR solution are depicted in Figure 5.

The reversible redox pair of peaks recorded at about −700–400 mV increased with the number of cycles indicating the accumulation of the polymeric product. The redox activity of the polymeric and monomeric forms of NR do not dramatically differ from each other and offer extended electron transduction in the surface layer [57]. It should be mentioned that the polymerization is initiated by rather high anodic potential (about 900 mV, this area is not shown on the voltammogram). Lower anodic potentials do not contribute to the formation of the polymeric coating.

**Figure 6.**
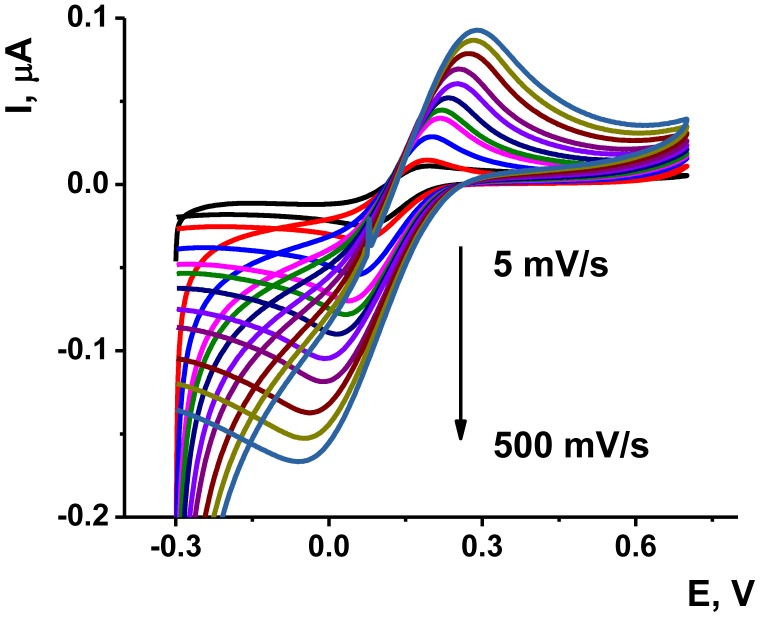
Cyclic voltammograms recorded on Au electrodes modified with poly-NR and Au/Boltorn H30^®^ suspension in the presence of 10 mM K_3_ [Fe(CN)_6_]. HEPES buffer solution, pH 7.0. Scan rate varied from 5 to 500 mV/s.

The deposition of the Au/Boltorn H30^®^ suspension on the poly-NR film improved the stability the signal examined in the presence of ferricyanide ion. The approriate voltammograms are presented on Figure 6. Although the polymerization was performed in phosphate buffer solution, the ferricyanide signals were studied in HEPES buffer solution, which is better compatible with the aptamers in voltammetric biosensors [55].

As could be seen from Figure 6, the ferricyanide peaks are well resolved from those of NR and no significant electron exchange in the surface layer takes place. The peak current *I_p_* linearly depends on the square root from the scan rate in accordance with Equation (1) for cathodic peak current. This indicates diffusion control of a charge transfer:

(1) $$I_{p},\mu A,=(10.3\pm 2.3)+(11.1\pm 0.3)\sqrt{v},mV/s,R^{2}=0.993,n=11$$

The kinetics of electron exchange and the influence of modifier were estimated from the dependence of the cathodic potential on the scan rate Equations (2) and (3) [56]:

(2) $$E_{p}=\frac{2.303RT}{\alpha n_{a}F}\log v+\text{Const}$$

(3) $$E_{p}=E^{0\prime }-\frac{RT}{\alpha n_{a}F}\left\lfloor 0.780+\ln\left(\frac{D^{1/2}}{k^{0}}\right)+\ln{\left(\frac{\alpha n_{a}Fv}{RT}\right)}^{1/2}\right\rfloor$$

Here *E*^0'^ is formal potential determined as half-sum of the peak potentials on voltammogram, *n_a_* is the number of electrons transferred in rate determining step (*n_a_* = 1 for ferricyanide ion), α is transfer coefficient, *D* is diffusion coefficient (7.6×10^−6^ cm^2^/s [58,59]) and *k*^0^ is heterogeneous electron transfer rate constant. The real working surface *A* accessible for the electron transfer was calculated from Equation (4) [45].

(4) $$I_{p}=2.99\times 10^{5}n{\left(\alpha n_{a}\right)}^{1/2}AD^{1/2}v^{1/2}c$$

where *c* is the concentration of ferricyanide ion. The characteristics obtained by Equations (2, 3, 4) are summarized in Table 1. Relative surface area is equal to the ratio of real and geometric working area.

As was mentioned above, anodization of the electrode improves the conditions for electron transfer. Deposition of Au/Boltorn H30^®^ suspension on the poly-NR film significantly increases the real surface ratio due to involvement of metal nanoparticles in the electron exchange. The *k_S_* value increases about three-fold against poly-NR and by about 16 times as compared with dendrimer layer. The latter one decreases also the real active surface probably due to partial blocking of the electrode area. The results presented for Au/Boltorn H30^®^ coating correspond to the maximal values obtained for the variation of the amounts of modifiers deposited onto the electrode. Both increase and decrease of this value (1 μL of suspension obtained from 0.71 g of Boltorn H30^®^ and 9 mg of AuCl_3_) resulted in worsening conditions of electron transfer, *i.e.*, decrease in the *k_S_* value and higher difference of the peak potentials of ferricyanide ions recorded by cyclic voltammetry. This can be related to the optimal coating of the electrode surface. Lower amounts of modifier leave part of the poly-NR film naked whereas higher amounts disturb direct mechanical content between the Au nanoparticles placed in non-conductive surrounding of Boltorn. Based on data on electron transduction obtained, the following experiments with aptamer immobilization and OTA measurements were performed with the above mentioned loading of a modifier.

### 3.3. OTA Determination

For aptamer immobilization, 5 μL of thiolated aptamer were carefully spread on the surface of electrode modified with poly-NR and Au/Boltorn H30^®^ suspension. The conditions for the surface layer assembling corresponded to those established for the best electron transfer with ferricyanide probe as described in Section 3.2. The covalent binding of thiolated aptamers on the surface of Au nanoparticles is simultaneous and irreversible. No evidence of the loss of aptamers from the Au surface was observed during the whole period of the aptasensor operation.

**Figure 7.**
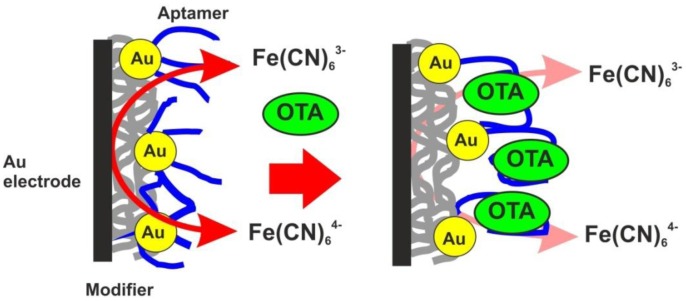
Principal scheme of the OTA signal generation with the aptasensor based on poly-NR and Au/Boltorn H30^®^ composite.

**Figure 8.**
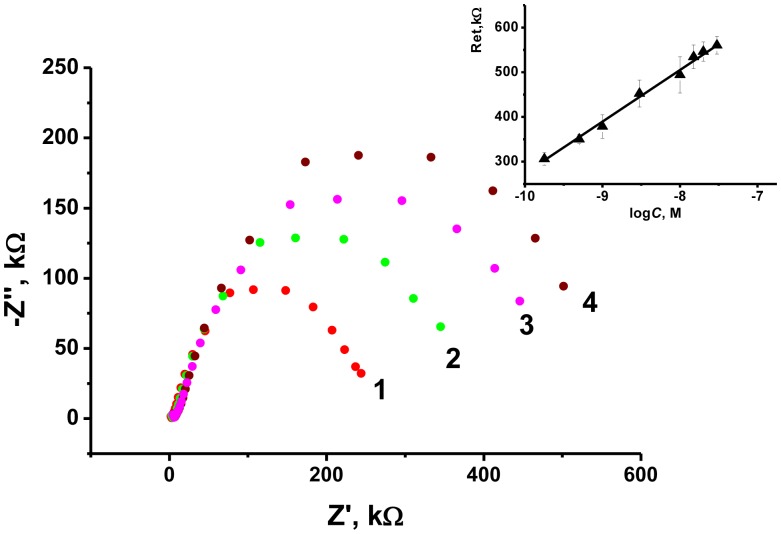
Nyquist diagrams of impedance spectra obtained prior to (1) and after addition of 1.0 (2), 10 (3) and 100 (4) nM OTA and the dependence of the charge transfer resistance on the logarithm of the OTA concentration (inset). Measurements in the presence of 0.01 M K_3_ [Fe(CN)_6_] and 0.01 M K_4_ [Fe(CN)_6_] at 0.235 V *vs*. Ag/AgCl. Frequency range 0.04 Hz–100 kHz, ac voltage amplitude: 5 mV.

As the transducer does not exert its own redox activity, the EIS mode was chosen for the OTA detection. The contact of aptamer with an analyte resulted in sharp increase of the charge transfer resistance due to conformational transfer of the aptamer from linear to guanine quadruplex and compaction of the surface layer. This decreases the access of the electrode surface for ferricyanide ions added as redox probe for EIS measurements. The general scheme of the aptamer function is shown in Figure 7. Figure 8 represents the Nyquist diagram obtained with various concentrations of an analyte. The resistance of the charge transfer, *R_et_*, linearly depends on the logarithm of the OTA concentration, logC_OTA_, in accordance with the Equation (5):

(5) $$R_{et},k\Omega =(732\pm 29)+(63\pm 2)\log C_{\text{OTA}},M,R^{2}=0.979,n=7$$

The limit of detection (LOD) calculated from S/N = 3 ratio was found to be 0.02 nM, whereas concentration range determined from 0.5 nM to 100 nM. Variation in the loading of modifier onto the electrode surface in ratio from 1:4 to 4:1 m/m from optimal amount resulted in decrease of the slope of calibration curve by 50%–75% and similar linearity area of calibration graph. The comparison with some other OTA biosensors reported is summarized in Table 2.

The LOD achieved in this work is lower than that of most aptasensors reported, except those applying magnetic separation and/or enzymatic amplification of the signal. However, in later case the assay is indirect and more complicated. The comparison of the results obtained with amperometric and impedimetric detection showed higher efficiency of EIS measurement which can be related to significant influence of surface conditions on charge transfer reactions. Besides them, electrochemilumenscent sensors offer comparable levels of analyte detection [41,66].

The aptasensors developed showed reproducibility of the *R_et_* value which ranges from 4.5% for single measurements performed with six aptasensors for 10 nM OTA to 7.5% for a series of six measurements with the same aptasensor within a weak operation. Commonly, the metrological characteristics obtained with the same sensor appear better than those of different sensors. In this case, the decrease of reproducibility is related to insufficient recovery of the aptasensor response. Treating the aptasensor with 0.1 M EDTA solution and 0.1 M NaCl did not lead to full recovery on the EIS characteristics. Instead, the response tends to decrease down to 80% recovery in six consecutive measurements. Meanwhile the storage of the aptasensors prepared prior to their application in dry conditions at 4 °C for at least two weeks did not alter the signal, while its variation increased during the storage period by 1.5–2.0 fold. For these reasons, the developed aptasensors can be recommended for a single use without any regeneration after their contact with the sample. Taking into account very low amounts of modifiers as well as simple preparation protocol, this does not lead significant increase in the measurement cost. As regards the Au/Boltorn H30^®^ suspension, it can be stored at 4 °C for at least one month without significant changes of the characteristics of the aptasensor prepared from it. Moderate improvement of the signal reproducibility can be achieved by sonication of the suspension prior to its use for 5–10 min. The procedure does not lead significant changes in the distribution of the Au nanoparticle size, but increases the uniformity of the sensing layer.

### 3.4. Selectivity and Real Sample Assay

The selectivity of the developed aptasensor was estimated under similar experimental conditions using an ochratoxin B standard solution. The slope of the calibration curve obtained in the concentration range from 1.0 nM to 100 nM was 25 kΩ/log*c*, or three times lower than that of OTA. The LOD of 1.0 nM makes it possible to detect at least 50-times higher concentration of the target analyte without any intereference and up to 50 nM with less than 10% deviation of the result. The maximal difference in the signals toward OTA and ochratoxin B were achieved for the OTA concentration of 10 nM. This is quite acceptable for direct detection of OTA in foodstuffs.

The application of Au nanoparticles as an aptamer carrier can interfere with some biological compounds, e.g., amino acids or thiols which are frequently present in the samples tested. However, no significant influence of 0.1 mM glycine, alanine, phenylalanine and cysteine added prior to or together with 10 nM OTA on charge transfer resistance was observed. The stability of the aptasensor signal can be referred to a strong interaction of Au nanoparticles with thiolated aptamer which is placed on their surface prior to contact with the sample. Rather dense coverage of the carrier surface with aptamer molecules prevents amino acids and thiols from their reaction with golden nanoparticles.

To confirm the prospects of the aptasensor in real sample assay, it was tested on the spiked samples of light and dark beer (“White Bear” and “Žateckў Gus Černý”, respectively). Prior to OTA spiking, the beer samples were boiled for 15 min until foaming stopped and then mixed with distilled water to their initial volume. The signal was measured in the conditions described for standard solutions. The recovery of about 70% for light beer and 78% for dark beer was obtained for six measurements with 5 nM OTA. Some losses of the analyte can be related to the OTA adsorption on solid particles remained in the beer. In HPLC experiments they are removed by filtration prior to OTA addition. The direct detection of OTA in undiluted beer accelerates testing and makes it possible to detect the OTA quantities below the maximal admissible levels established for foodstuffs [13,14].

## 4. Conclusions

In this work, an impedimrtric aptasensor has been developed for OTA detection on the base of novel aptamer carrier based on Au nanoparticles suspended in the dendrimeric hydrophilic polymer Boltorn H30^®^. Measurements of electrochemical properties of the modifier confirmed the high activity of Au nanoparticles in the electron transduction as well as improvement of the aptasensor characteristics in comparison with Boltorn H30^®^ and naked electrode. The use of the polymeric form of Neutral Red and thiolated aptamer against OTA made it possible to develop an easy protocol of aptamer immobilization and ensured the high sensitivity of the response. A LOD of 0.02 nM achieved under optimal conditions of biolayer assembly is lower than that of similar aptasensors with other signal transduction principles.

## Figures and Tables

**Table 1.** Electrochemical parameters of ferricyanide reduction on the Au electrode modified with various coatings. K_3_ [Fe(CN)_6_] 10 mM, HEPES buffer, pH 7.0.

**Electrode Pre-treatment**	**Modifier**	***k*^0^, cm/s**	**αn_a_**	**Relative Surface Area, %**
Anodization	Poly-NR	0.0025	0.45	110
No treatment	Poly-NR	0.0014	0.34	110
Anodization	Poly-NR—Au/Boltorn H30	0.0120	0.51	175
No treatment	Poly-NR—Au/Boltorn H30	0.0080	0.48	120
No treatment	Poly-NR—Boltorn H30	0.0005	0.33	80

**Table 2.** Analytical characteristics of electrochemical aptasensors for OTA detection.

**Signal Detection**	**Conc. Range, LOD**	**Reference**
Methylene Blue as a label	0.1–1000 pg/mL, LOD 0.095 pg/mL	[38]
Methylene blue as label, aptamer on Au nanoparticles	0.1–20 ng/mL	[60]
Tetramethylbenzidine oxidation in sandwich assay with peroxidase label	1–20 pg/mL, LOD 0.4 pg/mL	[61]
Hydroquinone oxidation in competitive assay with peroxidase and magnetic separation	0.78–8.74 ng/mL, LOD 0.07 ng/mL	[62]
Methylene Blue oxidation, indirect competitive assay with peroxidase label and magnetic separation	LOD 1.1 ng/mL	[63]
*R_et_*, aptamer chemisorbed on Au electrode	0.04–40 ng/mL, LOD 0.048–0.16 ng/mL	[64]
*R_et_*, aptamer immobilized on Ag nanoparticles	0.12–12 ng/mL, LOD 0.02 ng/mL	[65]
*R_et_*, aptamer immobilized on Au/Boltorn H30^®^ composite	0.4–40 ng/mL, LOD 8 pg/mL	This work
